# Supplementary material for: Are carbon emissions trading and green financial instruments synergistic? -Comprehensive quantitative research based on content analysis
Source: PLoS One. 2024 Mar 7;19(3):e0298601. doi: 10.1371/journal.pone.0298601 (PMC10919601; doi:10.1371/journal.pone.0298601)
Supplement: S1 Data — (ZIP) [file pone.0298601.s001.zip › Data/地级市Z_F工作报告.zip!/╣π╬≈/╣≤╕█╩╨/└┤╫╘╣1⁄2╓┌║┼í╛┬φ┐╦╩2╛▌═°í┐_2018.docx]

各位代表：

现在，我代表市人民政府，向大会报告政府工作，请予审议，并请市政协各位委员和列席会议的同志提出意见。

一、2017年工作回顾

过去一年，在自治区党委、政府和市委的坚强领导下，在市人大及其常委会和市政协的监督支持下，在市中级人民法院和市人民检察院的司法保障下，我们高举中国特色社会主义伟大旗帜，以马克思列宁主义、毛泽东思想、邓小平理论、“三个代表”重要思想、科学发展观、习近平新时代中国特色社会主义思想为指导，深入学习宣传贯彻党的十九大精神，围绕自治区持续营造“三大生态”，加快实现“两个建成”，扎实推进富民兴桂，奋力谱写新时代广西发展新篇章目标的部署，按照市委提出的“13446”工作思路，深化拓展“六个年”活动，持续开展“大招商、招大商”活动，凝心聚力，开拓创新，真抓实干，经济社会持续平稳健康发展，较好完成市五届人大二次会议确定的目标任务。

（一）经济发展稳中快进

预计全市地区生产总值突破千亿元，达1082.18亿元，增长9%；固定资产投资983.81亿元，增长16.9%，增速排名全区第一；财政收入90.03亿元，增长12.7%，增速排名全区第二；居民人均可支配收入20242元，增长8.6%。三次产业结构由19.8︰41︰39.2优化为17.9︰43︰39.1，第二产业占比提高2个百分点。

工业经济持续增长。深化拓展“工业发展优质年”“企业服务量化年”活动，工业实现扩量提质。规模以上工业总产值突破千亿元，达1182.25亿元，增长19.5%；规模以上工业增加值360.7亿元，增长12.2%，增速排名全区第二。新兴产业加速成长，产值增长145%，初步构建了广西第二汽车生产基地、中国­—东盟新能源电动车生产基地、电子信息制造基地和生物医药生产基地的框架。华奥新能源汽车投放市场，产值达15亿元；中国—东盟新能源电动车生产基地累计入驻企业75家、投产39家；赛尔康电子、战神专用车等项目投产，腾骏汽车项目进入设备安装阶段；新增自治区战略性新兴产业企业12家，排名全区第二。传统产业提档升级，冶金、机械制造、林板纸、纺织服装与皮革等产业产值增长20%以上。园区规模以上工业总产值759亿元，增长27.9%，占全市规模以上工业总产值的65%。

现代农业增产增效。农林牧渔业总产值334.75亿元，增长4.4%。粮食产量152万吨，粮食收购量连续四年位居全区第一。创建现代特色农业示范区47个，其中自治区级农业示范区5个。新增“双高”糖料蔗基地1.1万亩。荣获“中国生态富硒港”称号，累计建立富硒农产品生产基地88个，获认定富硒农产品32个、排名全区第一；新获中国名优（特色）硒产品6个、广西名优富硒产品3个。新增涉农商标113件，1013家涉农企业拥有自主品牌。新增农业龙头企业23家、农民专业合作社820家、家庭农场169家。主要农作物耕种收综合机械化水平60.1%。

现代服务业加快发展。社会消费品零售总额480.7亿元，增长11.3%；上亿国际汽贸博览城一期、美家居国际博览城一期运营，建成东湖农贸市场、铁路农贸市场和国旺农贸市场，苏湾现代综合物流园、西江综合物流信息平台加快推进；成功举办富硒农产品博览会、富硒百香果电商节、林产品展销会、汽车展销会等活动。港口货物吞吐量6322万吨，增长9.7%；集装箱22.2万标准箱，增长33.4%；促成了爱凯尔公司与北部湾港务集团的合作，推动了内河港口与沿海港口的强强联合、互动发展。接待游客总人数增长20%、旅游总消费增长29%；成功举办第四届中国汽车（房车）露营大会、中国健身名山登山赛；太平天国金田起义地址获评4A级旅游景区，新增3A级旅游景区6个、星级乡村旅游区及农家乐10家，港南区四季花田生态观光园获评广西五星级乡村旅游区，桂平市龙潭汽车旅游营地获评广西四星级汽车旅游营地。金融机构各项存款余额增长15.8%，贷款余额增长19.1%。餐饮、住宿、保险、快递等服务业稳步发展。

项目建设力度加大。深化拓展“项目建设丰收年”活动，推动项目多开工、多竣工，早开工、早竣工。主要建设项目完成投资355亿元，新开工项目205个、竣工115个，重大项目投资完成率排名全区第一。投资和项目建设全区领先，被选定为全区投资和项目建设工作会议主会场，在会上作经验介绍。列入国家重大市政工程领域PPP创新工作重点城市，郁江两岸综合治理工程、乡镇污水处理厂等PPP项目开工。储备“十三五”建设项目1882个，总投资3279.8亿元。建立投资项目网上预审平台，项目审批时限压缩了76%；实行重大项目集中预审，在项目启动阶段剔除“一票否决”因素，确保项目尽快落地。新增建设项目用地指标3万亩，盘活存量用地1.2万亩；推动农垦农场土地改革，解决贵港城市和产业发展用地瓶颈问题；土地集约节约利用成效好，获国家和自治区用地指标奖励6100亩。

县域经济快速发展。深化拓展“县域经济彰显年”活动，县域经济持续壮大，五个县市区地区生产总值增速均高于全区平均水平，桂平市、平南县、港南区、覃塘区规模以上工业增加值增速高于全区平均水平，平南县、港北区、港南区、覃塘区财政收入增速高于全区平均水平，桂平市、平南县、港北区、覃塘区固定资产投资增速高于全区平均水平。县域产业特色明显，桂平市纺织服装和机械制造产业迅速发展，平南县建材和饲料产业提档升级，港北区新能源电动车和电子信息产业集群初步凸显，港南区富硒农业、羽绒加工业、板材加工业不断壮大，覃塘区新能源汽车产业集聚效应显现。县域发展后劲增强，五个县市区共实施主要建设项目451个，完成投资额233.9亿元，分别占全市的58.3%和65.9%；县域招商成为招商引资的主力军，五个县市区共引进项目219个，合同投资额593亿元，分别占全市的69.5%和72.3%。

（二）改革创新深入推进

重点领域改革蹄疾步稳。“放管服”改革力度加大，清理取消行政许可事项54项、调整259项，提前实现政府部门权力清单和责任清单“两单融合”，率先在全区设立行政审批中介超市，“放管服”改革经验获《人民日报》推介。商事制度改革社会满意度、创业“便利度”排名全区第一，成为全国第三个、广西首个实施“口头申报、即可办照”简易登记模式的城市。组建了建投集团、交投集团、城投集团和金投集团，市场开发服务中心改革重组为市交投市场公司。全面启动城市公立医院综合改革，全部取消药品加成，实施药品零差率销售，探索实践医联体建设，县乡医疗服务一体化管理试点工作取得初步成效。供销社综合改革纵深推进，荣获“全国供销合作社综合改革试点工作先进单位”。全面落实河长制，建立了市县乡村四级河长体系。国有林场改革顺利完成，农村土地、城市执法、安全生产、养老服务、公共资源交易等领域改革持续深化。

开放合作上新台阶。2017年招商引资新签约项目315个、合同投资额819.8亿元，其中亿元以上项目208个，爱玛电动车、桥圩·中国羽绒谷、和乐门业等重大项目落户。截至2017年底，招商引资三年行动计划累计签约项目885个、合同投资额2283亿元，成功引进了华奥新能源汽车、腾骏汽车、赛尔康电子、嘉龙海杰电子、石家庄药业等一批大项目好项目，推动我市工业经济从“老五样”传统产业向“新三篇”新兴产业大飞跃。对外贸易规模扩大，进出口总额23.88亿元，增长26.6%；实际利用外资3405万美元，增长17%；贵港电子口岸和贵港口岸联检大楼投入使用，CEPA项目“绿色通道”开通。对外交流密切，与泰国洛坤府建立了国际友好城市关系。

创新驱动有新成效。获授权专利635件，新增国家高新技术企业12家、国家级星创天地2家，新建院士工作站2个，新增自治区级创新平台5个。建成科技企业孵化器9家，累计培育孵化企业155家。完成科技成果转化项目18项，任务完成率排名全区第一，市人民医院“医学3D打印技术在骨科修复重建的应用”获广西科技进步二等奖，泰亿诺公司获中国创新创业大赛“优秀企业”奖。新增自治区级以上质量品牌134个，广西水泥及建材产品质量检验中心、广西新能源电动车产品质量检验中心建设加快。

（三）城乡面貌日新月异

城市发展步伐加快。深化拓展“城市形象提升年”活动，实现“自治区文明城市”三连冠。城市总体规划修改全面启动，完成中心城区地下综合管廊、海绵城市、节水排水防涝设施等专项规划。开工建设城区至兴六高速公路一级连线拓宽工程、迎宾大道改造提升工程，贵港园博园即将竣工，罗泊湾大桥、青云大桥和同济大道通车，完成城区白改黑道路16条，改造易涝点30多个；完成城市楼宇、中山路北延段和城区公园等重点区域、重点路段的绿化美化亮化彩化。加快发展公交事业，入选国家“十三五”公交都市创建城市。大数据中心、云计算中心等9个智慧城市项目开工建设。市容市貌专项整治持续开展，城市“五乱”现象明显减少。机动车礼让斑马线成为文明交通新风尚。深入开展征地拆迁和“双违”整治大会战，“双违”现象得到遏制。城镇建设力度加大，桂平市“四路一桥”公共景观升级改造完成，平南县“一江两岸”主题景观带更加亮丽，木乐镇、桥圩镇入选全国特色小镇。常住人口城镇化率48.8%。

基础设施不断完善。贵港至合浦高速公路、梧州至柳州高速公路、来宾寺山至贵港公路、武林港疏港大道、港南木格至博白水鸣公路、桂平郁江二桥、港北高山大桥等建成通车。新建、改扩建农村公路594公里。贵港港苏湾作业区一期、武林作业区二期开港运营。投入6.97亿元，新建加固堤防8.6公里，完成渠道衬砌防渗113公里，新增节水灌溉面积6万亩，改善、恢复灌溉面积5.3万亩。升级改造低电压台区2017个。

生态环境有效改善。单位地区生产总值能耗下降3.5%，能耗总量控制在自治区下达目标任务内，万元规模以上工业增加值能耗下降7%。完成木材加工企业环境污染整改286家，淘汰黄标车和老旧机动车5850辆。完成全域永久基本农田划定工作。主要江河国控断面水质、县级以上集中式饮用水源地水质达标率100%。建成镇级污水处理设施10个，城区污水处理率92.1%。开展“美丽贵港”乡村建设活动，建成国家、自治区级绿色植保技术示范区7个，268家规模养殖场获“广西畜禽现代生态养殖场”称号；新建环保公厕49座，完成农村改厕4.7万户，荣获“全国厕所革命先进市”称号；完成农村厨房改造4.4万户；创建自治区级生态乡镇31个、生态村152个，排名全区第一。完成造林11.4万亩，森林覆盖率达46.3%。

（四）人民福祉持续改善

脱贫攻坚取得新成效。深化拓展“精准扶贫深化年”活动，预计65个贫困村、4.56万贫困人口摘帽脱贫。扶持贫困村发展特色种植7.1万亩、特色养殖42.8万头（羽）。修建贫困村屯道路1055公里，改造危房4468户，巩固提升饮水安全工程33个；完成易地扶贫搬迁项目17个，安置搬迁贫困群众9834人。发放1.5亿元资助贫困学生19.6万人次，科技特派员服务贫困村实现全覆盖。贫困人口基本医疗保险个人缴费比例由40%降低到20%，住院费用报销比例在2016年基础上再提高5%。有效衔接农村低保制度和扶贫开发政策，纳入低保贫困人口8.8万人。

民生保障日益完善。新增城镇就业1.8万人，农村劳动力新增转移就业4.6万人，城镇登记失业率1.11%。参加社会保险746万人次。企业、机关事业单位退休人员养老金分别提高6.5%和4.6%，城乡居民基本医疗保险补助标准提高7.1%。城乡最低生活保障标准分别提高12.9%和8.8%。投入8亿多元用于城乡低保救助、救灾、抚恤、保障残疾人和孤儿基本生活以及发放高龄津贴。养老服务水平稳步提升。基本建成保障性住房4523套，建设移民新村68个。

教育事业全面发展。西江教育园区建设加快，广西物流职业技术学院开工，贵港人民期盼已久的“大学梦”如愿起航。投入8.3亿元，新建和改造校舍36.5万平方米，建成桂平五中、桂平光明小学、木乐镇腾飞小学、平南镇东湖小学、港南一幼、港南二幼，开工建设贵港高中新校区、中山路小学等6所学校。学前三年毛入园率85.4%；义务教育巩固率95.1%，港北区、港南区通过国家义务教育均衡发展督导评估，平南县通过自治区评估；高中阶段毛入学率87.7%，每万人口高中在校生位居全区第一，高考上本科线人数位居全区第三。职业教育、民办教育、特殊教育等各类教育取得新成效。

文化体育事业更加繁荣。完成市文化艺术中心主体工程，新建102个村级公共服务中心。群众文化活动丰富多彩，“荷城大舞台”周周演深受群众喜爱。打造“荷文化”品牌成效明显，获得全国第32届 “荷花展”举办权。广播剧《红莲》获得广西第十四届精神文明建设“五个一工程”奖。贵县古墓葬群系列保护工作扎实推进。意识形态工作得到加强，社会主义核心价值观深入人心。全民健身和竞技体育全面发展，成功承办全国健美健身公开赛、广西第四届县域篮球赛和广西青少年男子篮球锦标赛。

卫生计生工作有新成绩。市妇幼保健院、儿童医院和妇产医院“三合一”业务大楼建成，港北区人民医院新业务综合楼投入使用。11家乡镇卫生院荣获国家“群众满意的乡镇卫生院”称号，乡镇和村级家庭医生签约服务覆盖率100%，村卫生室实施基本药物制度覆盖率96.4%。12家医院实现跨省异地就医直接结算服务。全面两孩政策稳妥实施，计划生育工作再上新台阶。

其他各项社会事业统筹发展。深入开展平安贵港创建活动，积极排查化解矛盾纠纷，依法打击违法犯罪，人民群众安全感满意度持续提升，荣获“建设平安广西活动先进市”称号。积极创建自治区卫生城市和食品安全示范城市，食品药品安全形势稳定向好。强化安全生产工作，安全生产事故和死亡人数实现“双下降”。民族宗教工作创新推进。在全区率先完成村（社区）“两委”换届选举工作。覃塘区大岭乡金沙村、覃塘镇龙凤村平田屯入选全国村民自治试点单位。国防动员、民兵预备役、人防和双拥等工作迈出新步伐，对台、侨务、统计、绩效、物价、编制、档案、地方志、地震、公积金、机关事务管理、二轻联社等工作取得新成效，工会、共青团、妇联、科协、文联、残联、贸促会、社科联、工商联和红十字会等群团组织在促进经济社会发展中发挥积极作用，海关、海事、检验检疫、农垦、邮政、通信、消防、水文、气象、烟草、金融、盐业、石油化工、船舶检验、地质勘探、无线电管理等中区直各部门和驻贵人民解放军、武警官兵为贵港经济社会发展做出新贡献。

（五）政府自身建设全面加强

认真学习宣传贯彻党的十九大精神，全面准确贯彻落实习近平新时代中国特色社会主义思想，切实增强“四个意识”，坚定“四个自信”，自觉在思想上政治上行动上同以习近平同志为核心的党中央保持高度一致。把全面从严治党贯穿于政府工作全过程，规范和加强党内政治生活。扎实推进“两学一做”学习教育常态化制度化。

坚持重大问题向市委请示报告，依法接受市人大及其常委会的监督，严格执行市人大及其常委会的决议决定，自觉接受市政协的民主监督，主动接受社会和舆论监督。坚持重大事项向市人大常委会报告、向市政协通报，积极听取各民主党派、工商联、无党派人士和人民团体的意见，人大代表建议和政协提案办结率100%。

加强法治政府建设，健全依法决策机制，严格规范行政执法行为，权力运行更加规范。加强立法工作，提请人大审议通过《贵港市太平天国金田起义遗址保护条例》。全面推进政务公开，政府部门权力清单和责任清单全部在政府网站公布。

持续开展“转作风、强责任、优服务、促规范”大行动，突出解决影响贵港发展的作风问题，大力弘扬敢干实干苦干善干的“四干”新作风，转变了政府职能，提高了行政效能。深入开展“市长服务企业接待日”“市政府服务接待日”活动，强化政府热线电话和微信平台建设，切实解决企业、市民和基层一线反映的困难问题。加快网上政府建设，强化网络问政，拓展贵港微政务平台，推进行政审批智能化试点，全面推行审批部门“说不”报告制度，建立招商引资预审服务机制，企业对贵港投资环境满意度位居全区第一。

认真落实中央八项规定和实施细则精神，严格执行国务院“约法三章”。进一步减文控会，文风会风持续改善。重点领域审计监督和行政监察得到加强。认真履行全面从严治党责任，加强党风廉政建设，推动政府系统廉洁从政。

各位代表！过去一年的成绩来之不易。这是自治区党委、政府正确领导和亲切关怀的结果，是市委统揽全局、科学决策的结果，是市人大及其常委会法律监督和市政协民主监督的结果，是市中级人民法院和市人民检察院司法保障的结果，是各级各部门和全市各族人民团结奋斗的结果。在此，我代表市人民政府，向全市各族人民表示崇高的敬意和衷心的感谢！向人大代表、政协委员，向人民法院和人民检察院，向各民主党派、工商联、无党派人士、各人民团体，向驻贵人民解放军、武警官兵，向中区直驻贵各单位，向所有关心支持贵港发展的社会各界人士表示衷心的感谢！

我们也清醒地看到，我市经济社会发展中还存在不少困难和问题。主要是：全方位发展不平衡不充分的问题依然突出，经济总量不大、质量不优的市情没有根本改变；加快发展的基础还比较脆弱，传统产业增长动力不足，新兴产业增长动能不强；制约发展的体制机制障碍还没有彻底打破，招商引资、深化改革任务依然艰巨；社会矛盾和问题交织叠加，社会建设和治理面临许多新情况新问题。政府工作仍存在不足，有些工作落实不够到位，少数干部担当不够，懒政怠政、推诿扯皮现象仍有发生。我们一定要直面挑战，敢于担当，下更大决心、想更多办法、用更大力度，全力以赴做好政府工作，推动各项事业再上新台阶。

二、2018年工作安排

2018年是全面贯彻落实党的十九大精神的开局之年，是改革开放40周年，是自治区成立60周年，是我市决胜全面建成小康社会、实施“十三五”规划承上启下的关键之年。党的十九大作出了中国特色社会主义进入了新时代、我国社会主要矛盾已经转化为人民日益增长的美好生活需要和不平衡不充分的发展之间的矛盾等重大政治论断，深刻阐述了新时代中国共产党的历史使命，确立了习近平新时代中国特色社会主义思想的历史地位，提出了新时代坚持和发展中国特色社会主义的基本方略，确定了决胜全面建成小康社会、开启全面建设社会主义现代化国家新征程的目标，对新时代推进中国特色社会主义伟大事业和党的建设新的伟大工程作出了全面部署。当前，经济形势尽管充满挑战，但总体来说机遇大于挑战。我们必须按照党的十九大作出的总体战略安排，把党的十九大提出的新思想、新理念、新要求贯穿于各项工作中，蓄力而发，乘势而上，努力开创贵港改革发展新局面。

今年政府工作总体要求是：全面贯彻落实党的十九大精神，以习近平新时代中国特色社会主义思想为指导，按照中央、自治区和市委的决策部署，坚持发展第一要务，全面贯彻新发展理念，紧扣社会主要矛盾变化，践行以人民为中心的发展思想，牢牢把握高质量发展的根本要求，围绕市委提出的“13446”工作思路，坚持“稳中求好、稳中快进”发展主基调，进一步深化细化“六个年”活动，保持经济高速度增长、高质量发展，加强和改善民生，全力打造新时代实力活力魅力给力新贵港，加快建成西江流域核心港口和新兴工业城市。

根据总体要求，今年经济和社会发展的主要预期目标是：地区生产总值增长8.5%，财政收入增长11%，规模以上工业增加值增长12%，固定资产投资增长13%，社会消费品零售总额增长10%，进出口总额增长10%，万元地区生产总值能耗下降3%，单位地区生产总值二氧化碳排放下降3.5%以上，万元规模以上工业增加值能耗下降3%以上，居民人均可支配收入增长8%，居民消费价格指数涨幅控制在3%左右，城镇登记失业率控制在4.3%以内，城镇新增就业1.6万人以上，常住人口城镇化率49 %以上，人口自然增长率控制在11‰以内。上述目标，主要是从充分体现高质量发展和稳中求好、稳中快进要求，积极稳妥兼顾需要与可能，衔接决胜全面建成小康社会和“十三五”发展目标等方面考虑的。

为实现上述目标，我们将重点做好以下几方面工作：

（一）加快结构调整，增强产业竞争力

做大做强工业经济。实施“工业提质增效年”“企业服务品牌年”活动，年内规模以上工业总产值增长20%以上，新增规模以上工业企业50家以上。打造新兴产业集群，确保华奥汽车二期、腾骏汽车、久久星整车生产线、嘉龙海杰电子等项目投产，推动赛尔康发展壮大，新兴产业产值占比15%以上。支持传统产业优化升级，加快贵钢特钢技改、贵糖技改搬迁等重点项目建设，推动建材、糖纸、机械、冶金等传统产业“二次创业”，加快桥圩·中国羽绒谷建设，促进传统产业向精深加工转型、产品向中高端提升。推进园区建设，规划建设自治区级电子信息产业园，深入开展园区基础设施建设大会战，加大土地收储力度，清理处置闲置土地和低效用地，提升园区承载力，年内园区规模以上工业总产值占全市规模以上工业总产值的70%以上。

推进农业现代化。实施农业产业提档升级行动，提高农业产业经营水平。稳定粮食生产，确保粮食产量150万吨以上。实施现代特色农业产业“10+3”提升行动，加强农产品商标品牌建设，培育农产品地理标志商标10个以上，新增“三品一标”品牌产品5个以上。大力发展大米、蔬菜、水果、茶叶等富硒特色农业，打响贵港百香果品牌，推进富硒农产品精深加工，加大富硒产品营销，提升“中国生态富硒港”影响力和竞争力。打造现代特色农业示范区升级版，建成各类示范区20个以上。建成8万亩“双高”糖料蔗基地。培育新型农业经营主体，新增农业龙头企业10家以上、农民专业合作社200家以上、家庭农场100家以上。扩大现代生态养殖，畜禽规模养殖场生态养殖比重60%以上。推进农业机械化，主要农作物耕种收综合机械化水平62%以上。发展休闲农业、农产品定制、“互联网+农业”等新业态，促进农村一二三产业融合发展。

提升现代服务业。实施现代服务业提速起飞行动，改造提升生产性服务业，积极发展生活性服务业，加快发展新兴服务业，促进服务业优化升级。加快现代物流园苏湾核心区、林产品交易物流园、羽绒交易中心和西江科技产业城等服务业集聚区建设。规划建设农贸市场，在中心城区新建农贸市场2个以上。规划建设贵港会展中心，举办具有贵港特色的各类大型展销会。发展港口运输业，港口货物吞吐量增长5%以上。大力发展红色旅游、生态旅游、乡村旅游、文化旅游和休闲度假旅游，接待游客总人数增长20%以上，旅游总消费增长30%以上；加快北环生态农业景观廊道、铜鼓湾温泉度假区、北山公园等项目建设，推动桂平西山风景区创建5A级景区，平天山国家森林公园、北帝山旅游区和雄森动物大世界创建4A级景区，启动桂平市创建国家全域旅游示范区、平南县和覃塘区创建广西全域旅游示范区工作。发展电子商务、金融保险、文化娱乐、健康、家政、教育培训、信息咨询、汽车服务等现代服务业。

（二）狠抓改革创新，释放发展新活力

深化重点领域改革。推进行政审批制度改革，加强行政权力运行监管和审批中介服务事项清理规范工作。整合部门各类热线电话服务，建设政府热线电话便民服务中心。完善国有资产管理体制和制度，推动国有企业规范运营。完善分级诊疗制度，建设县域医疗共同体，年内实现县域医疗共同体县级医院全覆盖，乡镇卫生院参与率50%以上。深化财政事权与支出责任划分改革，推动机关事业单位临聘人员管理改革，继续深化其他重点领域改革。

狠抓招商上项目。实施“项目建设效益年”活动，重点推进主要建设项目700个，完成投资400亿元以上。深化推进全员招商引资行动，招商引资实际到位资金增长10%以上，实际利用外资增长10%以上。强化产业链招商、集群招商，围绕做大“新三篇”、做强“老五样”，着力引进新能源汽车、电子信息、生物医药等新兴产业及其上下游项目，吸引建材、机械、冶金、羽绒、农产品加工等龙头企业或高端项目落户，打造一批特色鲜明、配套完善、成长性好的重点产业集群；继续“无中生有”，力争引进智能设备、机器人、电子芯片等重大新兴产业项目。坚持招大引强，面向粤港澳、长三角、京津冀和东北地区，瞄准世界500强、中国500强、民营企业500强和行业龙头企业精准招商，新签约亿元以上项目120个以上。

加强对外合作。积极参与珠江—西江经济带、粤桂黔高铁经济带和“一带一路”建设，加快融入北部湾经济区和粤港澳大湾区。实施加工贸易倍增计划，支持赛尔康、保利高等重点外贸企业做大做强。加快贵港西江保税物流中心（B型）建设，建成内河单一窗口。密切对外友好交流，推动包括贵港元素在内的中华文化走出去。

提升创新能力。年内每万人口发明专利拥有量增长28%以上，科技成果转化项目10个以上，新增国家高新技术企业9家以上。加大园区和企业研发经费投入，培育创新型龙头企业，发展壮大科技型中小企业。建设重点实验室和企业研发中心，推动新能源汽车和电动车、电子信息等领域技术创新。规划建设汽车零部件测试平台。加快贵港科技创业城、高新技术产业开发区、工程技术研究中心、农业科技园区、科技企业孵化器、众创空间和星创天地建设。支持企业与高校、科研院所合作，推动产学研一体化发展。实施高层次人才引进工程，大力引进高端人才、紧缺人才和创新团队。推进质量强市战略，提升贵港品牌的知名度和影响力。

（三）突出特色发展，增强县域经济实力

提振县域经济。实施乡村振兴战略，启动县域经济发展三年行动计划，深入开展“县域经济发力年”活动，年内五个县市区地区生产总值增速均高于全区平均水平，实现“广西科学发展先进县（城区）”或“进步县（城区）”零的突破。培育发展特色产业，桂平市重点发展机械制造、纺织服装等产业，做大做强旅游经济；平南县重点发展新型材料、保健食品、饲料等产业，力促建材产品向中高端升级；港北区重点发展新能源电动车、电子信息、生物医药等产业，推动糖纸、冶金、电力等传统产业提质增效；港南区重点发展电子信息、林产品加工、富硒农产品等产业，推动羽绒产业向精深加工转型；覃塘区重点发展新能源汽车、家具板材、精细化工等产业，壮大港口物流业。加强县市区产业园区规划建设，创建自治区级产业园区，推动产业向园区集聚，打造县域经济新增长极。

完善基础设施。加快建设贵港至隆安高速公路、荔浦至玉林高速公路、大安至镇隆一级路、覃塘至石卡产业园一级路、桥圩至东津公路、动界至寺面公路，建成根竹至平天山公路、金田起义景区公路，开工西外环高速公路和“九路两桥”项目。大力开展“四好农村路”建设，加快推进“镇镇通二级路、村村通双车道”，年内新建、改扩建农村公路350公里以上。完成贵港港总体规划修编，加快贵港电厂专用码头、保税中心码头和港区铁路建设，进一步完善江铁海联运体系。继续推进水利基础设施攻坚大会战，改善、恢复灌溉面积5万亩以上。加快大藤峡水利枢纽工程建设。

统筹城乡建设。实施县城发展提升工程，加强桂平市、平南县市政基础设施和公共配套设施建设，提升县城品位和承载力。加快特色小镇建设，把木乐镇打造成“中国休闲运动服饰小镇”，把桥圩镇打造成全国知名的“温暖小镇”，把大安镇打造成两广文化融合的民俗文化小镇，继续推进少数民族风情小镇建设。持续开展美丽贵港乡村建设，完成宜居乡村建设，启动幸福乡村活动。实施农村人居环境整治三年行动，推进“厕所革命”，建设清洁厨房，农村无公害化卫生厕所、清洁厨房普及率分别达到90%、60%。

加强服务扶持。指导各县市区做好发展规划，进一步明确发展思路、发展方向和发展着力点。落实行政审批下放事项，支持县市区先行先试，放开发展、放手发展。统筹资源配置，加大财政、用地、金融和人才的支持。加强分类指导，开展“一县一策”专项扶持，促进县市区发挥优势、加快赶超。完善考核机制，强化督查考评，激励先进、鞭策后进，形成攻坚克难有奖、干事创业光荣的鲜明导向和浓厚氛围。

（四）优化城市功能，提升城市品质

坚持规划引领。推进城市总体规划修改，加快形成西江流域核心港口城市和新兴工业城市互动融合发展的总体规划。完成港产城融合、商贸物流、城市综合交通等10项专题研究。加快新区规划建设，推动城区向西南方向发展，拉大城市框架。把覃塘区纳入中心城区规划建设。推进历史文化街区和历史建筑保护规划工作。加大控规编制工作力度，完成中心城区控规全覆盖。

完善城市设施。开展“城市发展跃升年”活动，实施城市建设“五大工程”，推进宜居城市建设，实现城市更净、更畅、更美、更亮。加强市政项目建设，完成城区至兴六高速公路一级连线道路拓宽工程、金港大道交通提升工程、解放路下穿铁路工程、高铁站前广场一期、西江大桥维修加固等项目，加快城区防洪工程、地下综合管廊一期、郁江两岸综合治理工程建设，开工建设高铁客运枢纽中心。继续实施城区道路“白改黑”工程。全面推进智慧城市建设，加快建设云计算中心、大数据中心、智慧政务、智慧城管、智慧消防、智慧交通、智慧市政、智慧公安等项目，推动城市规划、建设、管理加速向数字化、网络化、智能化发展。建设“海绵城市”，整治疏通河道，改造易涝点，基本实现城区道路雨后无积水。推进公交都市建设，建设公交专用道、公交充电站和公交首末站，增加公交线路和班次。深入实施城市绿化美化亮化彩化工程，提升城市品位。加快改造老城区，补齐基础设施和配套设施短板，焕发老城区活力。

强化城市管理。继续推进文明城市创建工作，全面提升城市管理水平。完善微信城管平台，鼓励市民参与城市管理，使城市管理问题能够第一时间发现、第一时间报告、第一时间解决。整合城市执法力量，改变城市管理“多龙治水”局面。开展市容市貌专项整治，突出解决交通拥堵、占道经营、电动车乱窜、黑车营运、车辆乱停乱放、渣土车滴撒抛漏、噪音扰民等“城市病”。保持打击“双违”高压态势，确保“双违”零增长、减存量。

（五）加强生态文明建设，建设美丽贵港

推进绿色发展。大力发展循环经济，加快粤桂（贵港）热电循环经济产业园、覃塘林业生态循环经济（核心）示范区建设，严格控制新上高耗能、高污染、高排放项目。倡导绿色低碳生活方式，开展创建节约型机关、绿色家庭、绿色学校、绿色社区和绿色出行等行动。

改善生态环境。坚守永久基本农田划定控制线，推进土地整治和高标准农田建设，确保耕地占补平衡。抓好水生态文明建设，强化落实河长制，推行湖长制，推进水资源管理和水土流失综合治理。完善污水处理设施，城区生活污水处理率93%以上。加强生态示范创建工作，力争港北区成功创建自治区级生态县（区），新增自治区级生态乡镇16个以上、生态村65个以上。加快创建国家森林城市，新增造林11万亩，森林覆盖率达到46.5%。

整治环境污染。打赢蓝天保卫战，持续开展大气污染防治行动，整治城市扬尘污染，淘汰黄标车和老旧机动车，开展工业企业全面达标排放综合整治行动，确保完成自治区下达城市环境空气质量改善目标。加强水污染防治，整治黑臭水体，确保主要江河断面水质和城市集中式饮用水源地水质达标。强化土壤污染管控和修复，改善土壤环境质量。创新环境监管执法，依法惩治环境违法行为。

（六）保障改善民生，增进群众获得感

优先发展教育事业。实施教育提升新三年行动计划，加快教育现代化。年内新建、改扩建公办幼儿园6所、中小学校13所、中职学校2所。加快西江教育园区建设，推进广西物流职业技术学院、广西工业学院、贵港高中新校区等项目，继续引进1~2所高等院校。实施第三期学前教育行动计划，学前三年毛入园率86%以上；推进义务教育均衡发展，义务教育巩固率95%以上，桂平市、平南县通过国家义务教育均衡发展督导评估。实施普及高中阶段教育攻坚计划，高中阶段毛入学率88%以上。大力发展职业教育，鼓励社会力量兴办教育，统筹发展其他各类教育。加强教师队伍建设，年内培养名校长100名、骨干教师（班主任）1000名，培训乡村教师10000名。

坚决打赢脱贫攻坚战。实施“精准扶贫突破年”活动，坚持精准扶贫、精准脱贫，力争年内74个贫困村摘帽、8.14万贫困人口脱贫。推进产业扶贫，探索“扶贫车间”模式，促进扶贫方式由“输血式”向“造血式”转变。加快易地扶贫搬迁，年内易地扶贫搬迁750户以上。修建贫困村屯道路500公里以上，加强贫困村饮水安全、农网改造、贫困户危房改造等工程建设。发挥贫困村资源优势，拓宽村集体经济增收新渠道。着力提升公共服务水平，实现贫困学生资助全覆盖，为贫困群众提供“先诊疗、后付费”和医疗扶贫“一站式”服务，加大科技、文化扶贫力度。注重扶贫与扶志、扶智相结合，激发贫困群众脱贫斗志和决心。

扩大社会就业。坚持就业优先战略，认真落实积极就业政策，努力实现更高质量和更充分就业。完善农民工创业园、创业孵化基地等平台，落实创业扶持政策，促进困难群众、返乡农民工、退役军人和高校毕业生等群体就业创业。加强职业技能培训，多渠道开发就业岗位，提供全方位公共就业服务。落实社保补贴、稳岗补贴等扶持政策，确保就业形势稳定。

强化民生保障。继续实施全民参保计划，扩大社保覆盖范围。建成市老年大学，推进市老年人养护院、养老服务园区建设。加强应急管理体系建设，提高综合防灾减灾救灾能力。完善社会救助、社会福利、慈善事业、优抚安置等制度，健全农村留守儿童、留守妇女和留守老年人关爱服务体系。发展残疾人事业，加强残疾康复服务。开工建设棚户区改造住房8000套以上，完成水库移民新村项目35个。

建设健康贵港。加快市中心医院建设，启动市中西医结合骨科医院整体搬迁，年内县乡医疗卫生机构基础设施全面达到国家标准。支持中医、壮瑶医事业发展。做好生育保健服务。推进自治区食品安全示范城市创建工作，加快市食品药品检验检测机构建设，开展食品药品安全专项整治行动，加强农贸市场农产品检验，把好群众饮食用药安全每一道关口。广泛开展全民健身活动，创建“健康贵港·运动休闲示范点”30个，积极申办国际、国家级重大体育赛事和全区青少年锦标赛。

发展文化事业。实施文化惠民工程，完善村级公共服务中心、农家书屋等文化设施。开展节庆文化活动，丰富群众文化生活。讲好贵港故事，创作一批反映新时代贵港题材的文艺作品。加强贵县古墓葬群保护，规范项目建设的文物勘探、发掘工作；做好大南门城楼、南江古码头、罗泊湾漪澜塔等文物修缮保护。全力办好全国第32届“荷花展”。严格落实意识形态工作责任制，不断提升意识形态工作能力水平。加强爱国主义教育，开工建设贵港市红色革命教育基地。培育和践行社会主义核心价值观，加强公民道德和精神文明建设，全面开展创建文明镇村（社区）工作，争创一批全国和自治区级文明村镇。

创新社会治理。完善社区治理体系，推动社会组织管理体制创新。开展法律服务惠民工作，构建覆盖城乡公共法律服务体系。建设更高水平的平安贵港，做好信访和人民调解工作；加快推进“雪亮”工程项目，完善立体化、信息化社会治安防控体系，打造共建共治共享的社会治理格局；强化反恐维稳，依法打击和惩治黑拐枪、盗抢骗、黄赌毒等违法犯罪活动。加强诚信建设，营造诚信社会环境。落实安全生产责任制，深入开展安全风险管控和隐患排查治理，坚决防范重特大事故。

统筹抓好其他工作。加大民族地区发展支持力度，依法管理宗教事务。加强国防动员和后备力量建设，加大双拥创建工作力度，抓好军民融合发展，创建新一轮全国、全区双拥模范城。开工建设市民中心、城市党建综合服务中心。继续推进自治区卫生城市创建工作，抓好档案、统计、公积金、粮食、物价、编制、人防、地方志、地震、绩效、二轻联社、机关事务管理、对台和侨务等工作，充分发挥工会、共青团、妇联、科协、文联、残联、贸促会、社科联、工商联和红十字会等群团组织作用，支持海关、海事、检验检疫、农垦、邮政、通信、消防、水文、气象、石油化工、烟草、电力、金融、盐业、船舶检验、地质勘探、无线电管理等中区直部门工作。

三、全面加强政府自身建设

各位代表！新时代意味着新起点新要求，新时代呼唤着新气象新作为。我们将持续加强和改进政府工作，坚持实字当头、干字为先，直面问题、说到做到，努力建设人民满意的服务型政府。

提高政治能力。把讲政治摆在第一位，增强“四个意识”、坚定“四个自信”，坚决维护习近平总书记在党中央、全党的核心地位，坚决维护以习近平同志为核心的党中央权威和集中统一领导，坚决在思想上政治上行动上同以习近平同志为核心的党中央保持高度一致，坚决贯彻落实党中央、国务院和自治区党委、政府以及市委决策部署。以全面从严治党统领政府各项工作，扎实推进“两学一做”学习教育常态化制度化，开展“不忘初心、牢记使命”主题教育，自觉用习近平新时代中国特色社会主义思想武装头脑、指导实践、推动工作。

坚持厉行法治。推进依法行政，建设法治政府，严格规范公正文明执法，认真做好行政复议应诉工作。坚持科学决策、民主决策、依法决策，严格落实重大行政决策法定程序。做好《贵港市城市道路管理条例》《贵港市城市市容和城市卫生管理条例》等地方性法规的起草工作。依法接受市人大及其常委会的监督，自觉接受市政协的民主监督，主动接受社会和舆论监督，认真听取人大代表、政协委员、民主党派、工商联、无党派人士和各人民团体的意见建议，办好人大代表建议和政协提案。提高政务公开制度化标准化水平，保障公众知情权、参与权。

强化工作落实。增强狠抓落实本领，坚持说实话、谋实事、出实招、求实效，把雷厉风行和久久为功有机结合起来，勇于攻坚克难，以钉钉子精神做实做细做好各项工作。落实工作责任制，按照目标明确、措施明确、阶段性目标明确、责任人明确的要求，推动各项工作具体到单位、实化到项目、量化到指标；坚持问题导向，做到及时主动发现问题、及时主动解决问题、及时主动协调问题、及时主动报告问题；建立健全督促检查机制，抓好决策部署、重大政策、重大项目、重大工程的落实。

加强作风建设。认真贯彻落实中央八项规定和实施细则精神，严格执行党风廉政建设责任制和廉政准则，切实把政府系统党风廉政建设推向深入。强化审计监督，实行公共资金、国有资产、国有资源和领导干部经济责任审计全覆盖。大兴调查研究之风，扑下身子，沉到一线，紧扣人民群众生产生活，紧扣经济社会发展实际，了解情况，研究问题，踏踏实实干好工作，力戒形式主义、官僚主义。加强正风肃纪，重拳整治不作为、慢作为、乱作为，坚决打赢作风建设攻坚战、持久战。

各位代表！实干成就梦想，拼搏创造未来。我们要更紧密地团结在以习近平同志为核心的党中央周围，坚持以习近平新时代中国特色社会主义思想为指引，在自治区党委、政府和市委的坚强领导下，不忘初心，牢记使命，砥砺前行，锐意进取，为打造新时代实力活力魅力给力新贵港，加快建成西江流域核心港口和新兴工业城市而努力奋斗！
